# Effects of a neuromuscular training program on physical performance and asymmetries in female soccer

**DOI:** 10.3389/fphys.2023.1171636

**Published:** 2023-05-02

**Authors:** Alberto Roso-Moliner, Elena Mainer-Pardos, Antonio Cartón-Llorente, Hadi Nobari, Svein Arne Pettersen, Demetrio Lozano

**Affiliations:** ^1^ Health Sciences Faculty, Universidad San Jorge, Zaragoza, Spain; ^2^ Faculty of Sport Sciences, University of Extremadura, Cáceres, Spain; ^3^ School of Sports Sciences, UiT the Arctic University of Norway, Tromsø, Norway

**Keywords:** soccer (football), intervention, physical performance, interlimb asymmetry, strength, power, dynamic balance

## Abstract

**Introduction:** Women’s football require optimal neuromuscular system development for injury prevention and performance optimization. Standardized neuromuscular training programs have shown promising results in reducing injuries and functional asymmetries, but evidence on their impact on performance is limited.

**Methods:** This research examined the effects of a 10-week neuromuscular training program on physical performance and asymmetries in female football players. Thirty-eight female players from two Spanish Second Division women’s football teams participated in the study. The physical performance tests used were: ankle dorsiflexion, bilateral and unilateral horizontal jump, bilateral and unilateral vertical countermovement jump, 40 m sprint including partial times at 10, 20 and 30 m and the 505 test for change of direction evaluation. For 10 weeks, players in the experimental group performed three weekly 24-min neuromuscular training sessions. Participants in the control group completed their normal 24-min strength and conditioning program.

**Results:** The main results were that maximal linear velocity and change of direction skills showed the most notable improvements [effect size (ES), 0.46 to 0.59] after implementation of the training program, ankle dorsiflexion and jumping skills, also improved although, to a lesser extent (ES, <0.35) while asymmetries between limbs were reduced. Maximal running speed improved in the intervention group (*p* < 0.001) with a mean ES −0.59.

**Discussion:** We conclude that a 10-week neuromuscular training program can be a sufficient stimulus to improve football-specific performance variables in high-level female football players. Therefore, female players and coaches should be aware that weekly inclusion of strength, power and dynamic balance exercises following a neuromuscular paradigm is helpful for football-specific performance improvement.

## 1 Introduction

Football is currently one of the most popular sports worldwide, attracting enormous media and commercial interest. Notably, women’s football is experiencing dramatic growth in recent years, reflected in a continuous increase in female players each year (Association, 2007). It is spreading throughout many countries all over the globe through international and local promotion programs (Association, 2020). At the same time, this media and economic momentum have led to a refinement in-game analysis and improved training methods for performance optimization and injury prevention through scientific knowledge.

Regarding its physiological demands, football is an intermittent sport in which an average of 150–250 short, high-intensity actions are performed during the 90 min of the game (Mohr et al., 2003). The average distance covered during a match in women´s football ranges between –8,200–11,000 m among professional-level players, slightly higher in international competitions and minimally lower in collegiate players (Winther et al., 2022). For an exemplary implementation of these demands, cardiorespiratory capacity is a fundamental aspect of the players’ physical condition (Stolen et al., 2005). However, football also requires coping with a large number of short and repeated high-intensity actions, such as shooting, sprinting, jumping, accelerating and decelerating, often including change of direction (COD), crucial determinantes of success or failure in the game, and also good predictors of players’ performance level (Lockie et al., 2018).

Football-specific high-intensity actions add substantional physiological stress to the players, including the anaerobic and neuromuscular systems (Datson et al., 2014). Moreover, a study by Faude et al. (2012) confirmed that jumping, cutting, and sprinting generate more than 50% of inciting events that end as high-speed impacts with the opponent or intrinsic musculoskeletal injuries, underlining the relevance of preparing for these actions not only from a performance aspect (Pardos-Mainer et al., 2019) but also for injury prevention (Wright and Laas, 2016). In this regard, previous studies have investigated the existence of functional imbalances and lower limb asymmetries in women´s football through a field test (Pardos-Mainer et al., 2019; Pardos-Mainer et al., 2020), and others found that functional asymmetries not only lead to an increased likelihood of injury but also a decreased performance (Menzel et al., 2013; Lockie et al., 2014) Different valid physical tests such us unilateral jump tests including the vertical jump (i.e., the countermovement jump (CMJ)) and the horizontal jump have been considered in scientific studies to reflect functional asymmetries in football (Bishop et al., 2018; Bettariga et al., 2022).

Considering the relevance of repeated short, high-intensity actions in football performance and injury occurrence, a wide variety of standardized neuromuscular training programs look for optimal neuromuscular system development. These multicomponent protocols typically combine mobility, stability, plyometric strength, and COD exercises to enhance neuromuscular coordination and motor control in a sport-specific environment. Among them, the Sportsmetrics^TM^, Harmoknee and FIFA 11+ protocols have already shown promising results in reducing injuries in male and female football players (Noyes et al., 2013). Furthermore, a recent systematic review and meta-analysis highlight the effects of neuromuscular training interventions on functional asymmetries in football, suggesting that reducing them could lead to a decreased risk of injury and improved performance (Bettariga et al., 2022).

Despite, the promising results of neuromuscular training programs on asymmetries and injuries, scientific evidence on the effects of these interventions on performance remains scarce. The limited number of studies that investigated the impact of these programs on football performance reported improvements in linear speed, jumping and COD ability (Noyes et al., 2013; Pardos-Mainer et al., 2019; Liu et al., 2021). In contrast, other studies that included part of, but not all, the components of a neuromuscular training program showed concurrent results (Pardos-Mainer et al., 2020; Pardos-Mainer et al., 2021).

Notwithstanding, the growing evidence on the effects of neuromuscular training in football, their role in improving critical performance skills remains unclear, and more studies are needed to understand better the potential of these training programs, particularly in women´s football. Therefore, the purpose of the present study was to evaluate the effects of a neuromuscular training program on physical performance and asymmetries in female football players. Based on the scientific literature (Pardos-Mainer et al., 2019; Pardos-Mainer et al., 2020; Roso-Moliner et al., 2022), we hypothesized that a 10 week neuromuscular intervention increases physical performance and reduces asymmetries in female football players.

## 2 Materials and methods

### 2.1 Participants

**FIGURE 1:**
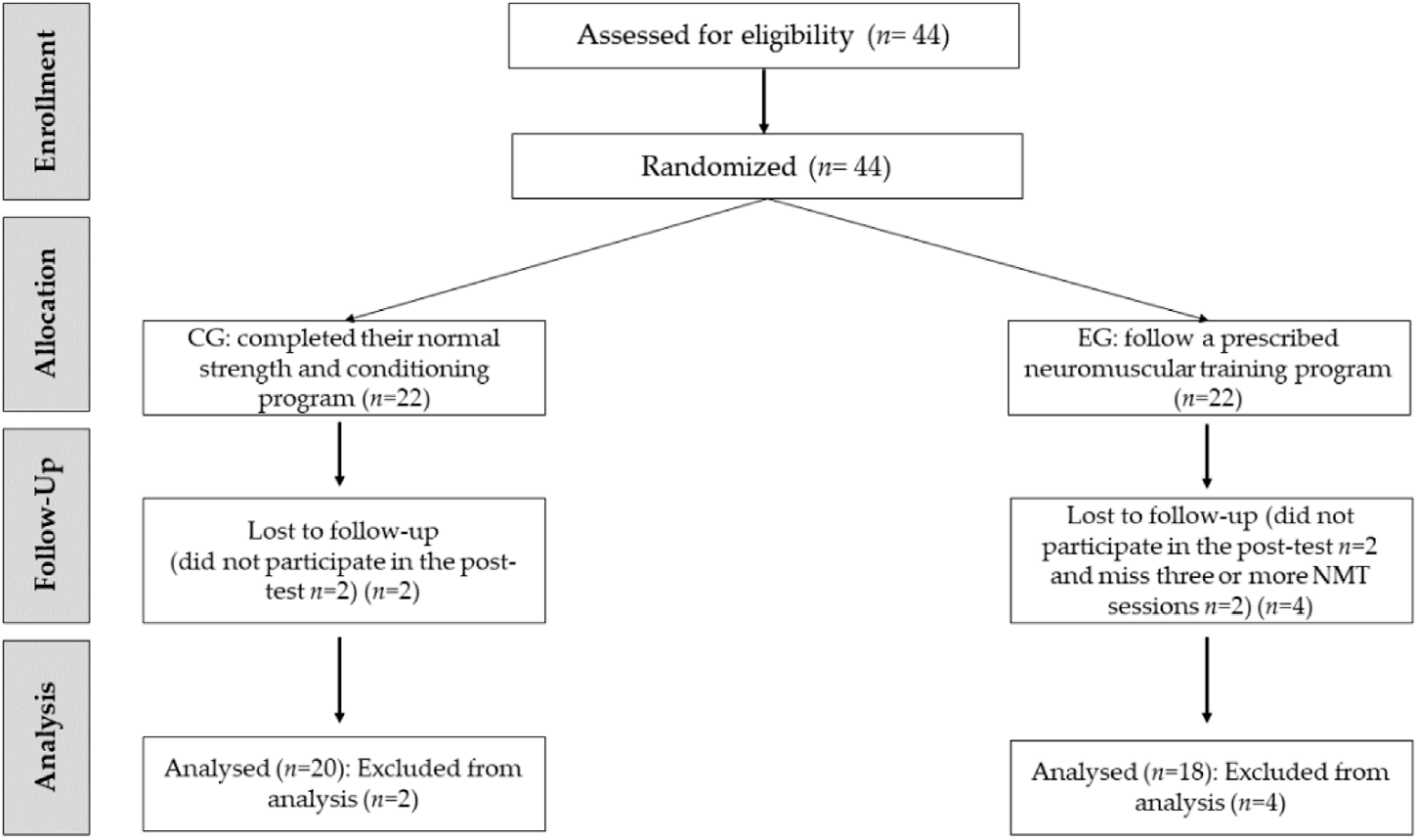
Recruitment process described in a CONSORT diagram.

Thirty-eight female football players from two Spanish Women’s Second Division teams participated in the current study. Both teams followed a weekly football training regimen comparable in volume and methodology (five sessions lasting 90 min and one match each week). All participants met the following inclusion criteria: 1) minimum 6 years of football training and competition experience; 2) regular football training and competition for 6 months prior to data collection; 3) being injury-free for at least 3 months; and 4) not participating in other NMT or diet programmes outside of this study. In addition, the following exclusion criteria were met 1) missing three or more NMT sessions or 2) missing one test day. Data collection started in the seventh month of the competitive season. The players were randomly assigned to an experimental group (E.G., n = 22) or a control group (CG, n = 22) (Figure 1). All participants signed informed consent, and the ethical standards of the Declaration of Helsinki were followed. The study was approved by the Local Clinical Research Ethics Committee (PI21/011, CEICA, Spain).

*A priori* sample size calculation was performed using G-Power software Version 3.1.9.7 (University of Dusseldorf, Dusseldorf, Germany) with the following specifications: F tests through ANCOVA with fixed effects, main effects, and interactions, effect size f = 0.50 based on previous studies (Zouhal et al., 2019; Isla et al., 2021; Nunes et al., 2021; Brini et al., 2023), *α* err prob = 0.05, power (1-β err prob) = 0.85, number of groups = 2, and numerator df = 1. The calculated sample size of 38 participants was found to provide an 85.01% chance of successfully rejecting the null hypothesis of no difference in the variables studied.

### 2.2 Exercise protocol

All participants trained in the same schedule of football training. The, EG performed four familiarization sessions to practice the neuromuscular training routine 2 weeks before the start of the intervention. Before the data collection, all players did a warm-up of the lifting, activation, mobilization, and potentiation (RAMP) (Jeffreys, 2006). For 10 weeks, female players in the, EG performed three 24-minute neuromuscular training sessions per week. Control group participants completed their normal conditioning routine (i.e., mobility and strength exercises) at the same time. Neuromuscular training intervention included mobility (lunge to hamstrings dynamic stretch, standing hip out, 90–90 hip stretch), stability (star excursion balance, side jumps + balance, forward hop + balance), anterior chain strength (squat, squat jump, and walking lunge), lumbo-pelvic control (front, side, and add plank), posterior chain strength (1 leg glute bridge, 1 leg touch and hop, scissors lunge) and change of direction (lateral shuffle, t-test, 505 test). Each training session consisted of 6 exercises (one for each category mentioned above) performed as a circuit (i.e., four sets with a work-to-rest ratio of 40:20 s). The working leg was switched during each set for unilateral exercises. As previously suggested (Roso-Moliner et al., 2022), the selected exercises progressed in load and specificity during the intervention. Thus, level 1 activities were completed during the first 2 weeks and were upgraded to level 2 and 3 exercises in weeks 3 and 7, respectively. Additionally, a modified Borg scale (score from 0 to 10) (Zamunér et al., 2011) was used to individually monitor the perceived intensity of the training sessions in both groups.

#### 2.2.1 Performance measurements

Pre- and post-tests were performed during the first days of the week before and after the intervention (17:00–19:00 h, same environmental conditions: –22°C and –20% humidity). Participants were instructed to abstain from vigorous exercise for at least 48 h before data collection, and all tests were carried out on a football field with football boots. In addition, to avoid possible confounding effects of diet on performance assessment, a registered dietician-nutritionist performed a 24-hour food recall on the test days and calculated the mean macronutrient and energy intake (DAPA Measurement Toolkit, Cambridge, UK). The Spanish Database of Food Composition (BEDCA) was used to analyse the information from these 24-hour food recalls (Roso-Moliner et al., 2022).

#### 2.2.2 Ankle dorsiflexion range of motion

The LegMotion system (LegMotion, your Motion, Albacete, Spain) was used to assess the ankle dorsiflexion range of motion (ROM), and the test is described elsewhere (Pardos-Mainer et al., 2019). Each player performed three trials with each ankle to make the most appropriate measurement, recovering 10 s between each attempt. The intra-class correlation (ICC) was 0.86 in this test.

#### 2.2.3 Horizontal jump test

The bilateral and unilateral horizontal power (unilateral and bilateral) was measured with the horizontal jump. A standard tape measure (30m M1; Stanley, New Britain, United States) was used to measure this test, described elsewhere (Pardos-Mainer et al., 2017). After two attempts, the best jump was recorded for future analysis, with a 60-second recuperation between each jump. The ICC was 0.84 and 0.88 in the bilateral and unilateral horizontal power.

#### 2.2.4 Countermovement jump test

The CMJ was used to measure both bilateral and unilateral vertical jumping ability. Optojump (Optojump, Microgate, Bolzano, Italy) was used to determine jump height (Pardos-Mainer et al., 2017). The test was repeated three times, with at least a 45-second passive recovery period between each, and the best jump was registered for further analysis. The ICC was 0.89 and 0.90 in bilateral and unilateral horizontal power.

#### 2.2.5 40-metre speed test

The sprint speed was assessed through 40-m sprint test with 10-, 20-, and 30-m split times. Total and partial times were measured using dual beam photocell systems (Witty, Microgate, Bolzano, Italy) placed 1 m above ground level at the abovementioned marks. All participants started standing once ready and 0.5 m behind the first photocell. The test was performed twice, separated by at least 3 min of passive recovery, and the best time was recorded for analysis. The ICC value was 0.93.

#### 2.2.6 505 change of direction test

The agility was assessed using the 505 COD test with dual beam photocell systems placed 1 m above the ground level (Witty, Microgate, Bolzano, Italy) and performed as Spiteri et al. described (Spiteri et al., 2015). Players build up speed for 10 m, and as they pass through the electronic timing system, they sprint 5 m, make a 180º turn and sprint 5 m again. Each leg (right and left) completed the test twice, with at least three minutes of passive recovery in between, and the best time was recorded for analysis. The most effective time for analysis was noted. The ICC value was 0.84.

### 2.3 Statistical analysis

The normality of all variables was checked through the Shapiro-Wilk test. Relative reliability analysis was examined by the intra-class correlation coefficient (ICC). We have analysed covariance (ANCOVA) analysis to compare between groups, considering pre-test as the covariate and reported partial eta (ηp^2^) ES. If the results of both groups were comparable, progress was compared using a *t*-test and percentage change. The standardized mean difference (Hedges’ *g*), representing ESs, is shown along with 95% confidence intervals (CI). The categories of trivial (0.2), small (>0.2), moderate (>0.5), and large (>0.8) were used to classify the ES. The ESs were interpreted using Hopkins et al. guideline’s for the standardized mean difference to determine the number of pairwise comparisons between the pre- and post-test (Hopkins et al., 2009). The significance of statistical analysis was considered when *p* < 0.05. All tests and statistical calculations were performed with the SPSS program (version 28.0, IBM SPSS Inc. Chicago, IL, United States).

## 3 Results

No significant differences (*p* = 0.45) were found during the intervention in the modified Borg scale of perceived exertion (CG: 7.26 ± 0.23; EG: 7.3 ± 0.25).

The ANCOVA results showed no significant group by time interactions of the improvement pre- and post-test in ROM and percentage asymmetry tests. Table 1 and Figure 1 illustrate the percentage changes in ROM variables between the pre- and post-test.

**TABLE 1:** Results of the range of motion variables in the control and experimental group.

Variables (cm)	CG (n = 20)					EG (n = 18)			
	Pre-intervention (mean ± SD)	Post-intervention (mean ± SD)	Pre-post (%)	ES (95% CI)	Pre-intervention (mean ± SD)	Post-intervention (mean ± SD)	Pre-post (%)	ES (95% CI)
ROM R	44.02 ± 4.31	44.32 ± 4.17	0.68	0.07 (-0.55; 0.69) T	41.09 ± 4.59	41.66 ± 4.35	1.38	0.12 (-0.53;0.77) T	
ROM L	43.75 ± 4.97	44.22 ± 5.19	1.07	0.09 (-0.53; 0.71) T	40.03 ± 4.68	40.69 ± 4.66	1.64	0.13 (-0.52;0.79) T	
% As ROM	4.16 ± 4.68	5.37 ± 4.53	29.24	0.25 (-0.37; 0.87) S	3.96 ± 3.77	3.81 ± 3.62	−3.97	−0.04 (-0.69; 0.61) T	

CG, control group; EG, experimental group; SD, standard deviation; ROM, range of motion; R, right; L, left % As: percentage of asymmetry; ES, effect size; CI, confident interval; T, trivial; S: small, *p< 0.05.

**FIGURE 2:**
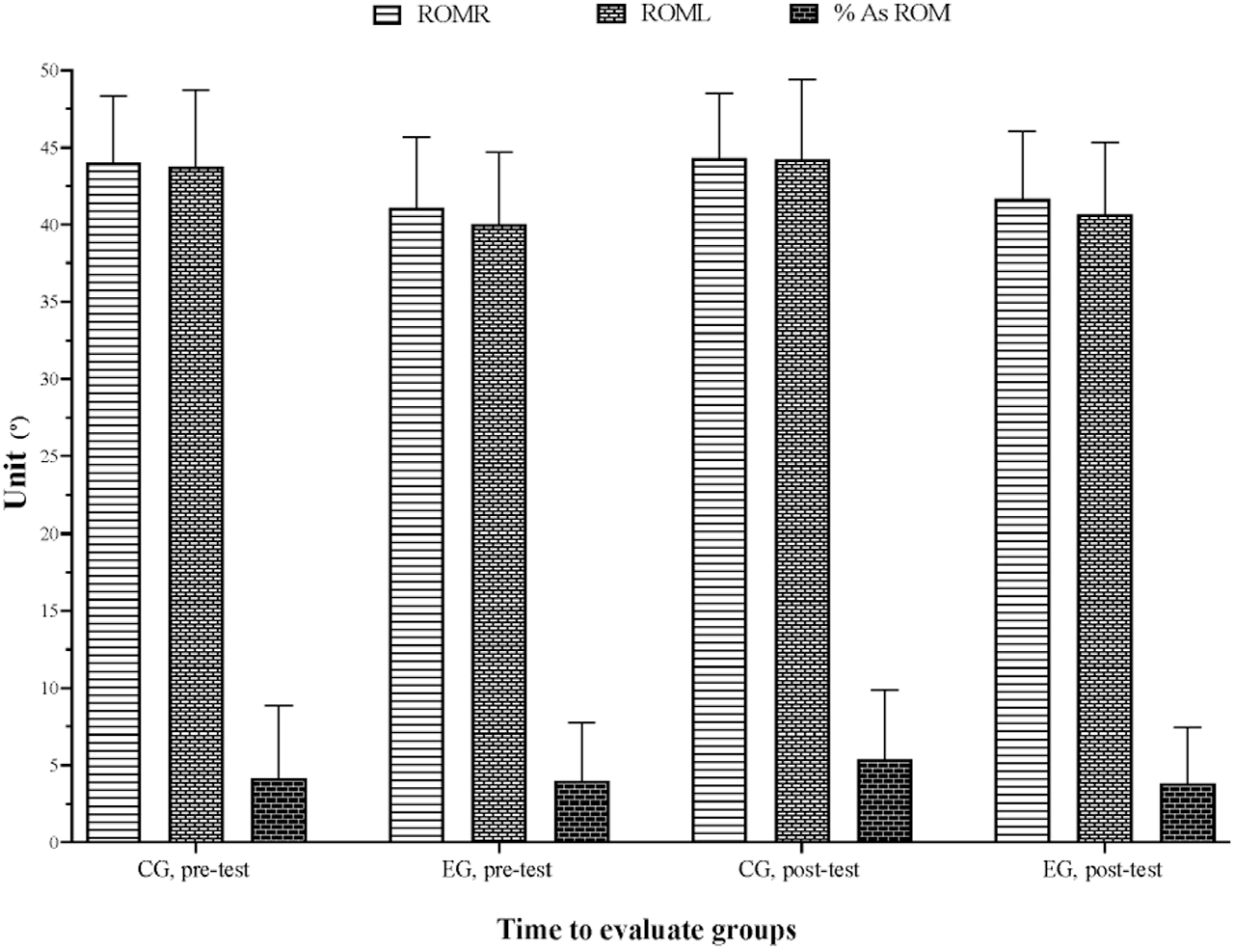
Variation in ROM variables for control and experimental group.

The ANCOVA results showed significant group by time interactions for CMJ (*p* ≤ 0.001, f = 38.777, ηp^2^ = 0.526), CMJ right (*p* ≤ 0.001, f = 30.455, ηp^2^ = 0.465), CMJ left (*p* ≤ 0.001, f = 38.777, ηp^2^ = 0.388) and HJ (*p* ≤ 0.001, f = 17.137, ηp^2^ = 0.329). When comparing the percent change from pre-to post-intervention in HJ, the independent groups *t*-test revealed a significant difference between, EG and CG (*p* = 0.001). HJ in the, EG increased by 0.55% compared to −0.07% in CG. Table 2 and Figure 2 illustrate the percentage changes in jump variables between the pre- and post-test.

**TABLE 2:** Results of vertical and horizontal jump variables in control and experimental group.

Variables (cm)	CG (n = 20)				EG (n = 18)			
	Pre-intervention (mean ± SD)	Post-intervention (mean ± SD)	Pre-post (%)	ES (95% CI)	Pre-intervention (mean ± SD)	Post-intervention (mean ± SD)	Pre-post (%)	ES (95% CI)
HJ	168.10 ± 10.34	167.98 ± 10.27	−0.07	−0.01 (−0.63; 0.61) T	179.99 ± 9.00	180.97 ± 8.78	0.55	0.10 (−0.55;0.76) T
HJ R	141.26 ± 11.88	141.33 ± 12.29	0.52	0.01 (−0.61; 0.63) T	152.19 ± 9.92	152.43 ± 9.45	0.16	0.02 (−0.63;0.68) T
HJ L	141.88 ± 10.66	142.62 ± 11.31	0.05	0.06 (−0.56; 0.68) T	153.07 ± 8.86	153.82 ± 9.23	0.49	0.08 (−0.57;0.73) T
% As HJ	2.46 ± 1.67	2.58 ± 1.57	4.88	0.07 (−0.55; 0.69) T	2.31 ± 1.94	2.29 ± 1.71	−0.87	−0.01 (−0.66;0.64) T
CMJ	28.23 ± 2.09	28.25 ± 2.16	0.07	0.01 (−0.61; 0.63) T	27.19 ± 10.34	27.73 ± 10.34	1.95	0.23 (−0.43;0.88) S
CMJ R	13.86 ± 1.63	13.82 ± 1.66	−0.31	−0.02 (−0.64; 0.60) T	13.17 ± 10.34	13.67 ± 10.34	3.78	0.25 (−0.41;0.91) S
CMJ L	13.85 ± 1.21	13.81 ± 1.20	−0.27	−0.03 (−0.65; 0.59) T	13.15 ± 10.34	13.53 ± 10.34	2.84	0.20 (−0.45;0.86) S
% As CMJ	5.61 ± 4.97	5.50 ± 4.98	−1.90	−0.02 (−0.64; 0.60) T	5.22 ± 10.34	5.09 ± 10.34	−2.37	−0.02 (−0.68;0.63) T

CG, control group; EG, experimental group; SD, standard deviation; ES, effect size; CI, confident interval; T: trivial; S, small; HJ, horizontal jump; R, right; L, left; CMJ, countermovement jump; % As: percentage of asymmetry; *p < 0.05.

**FIGURE 3:**
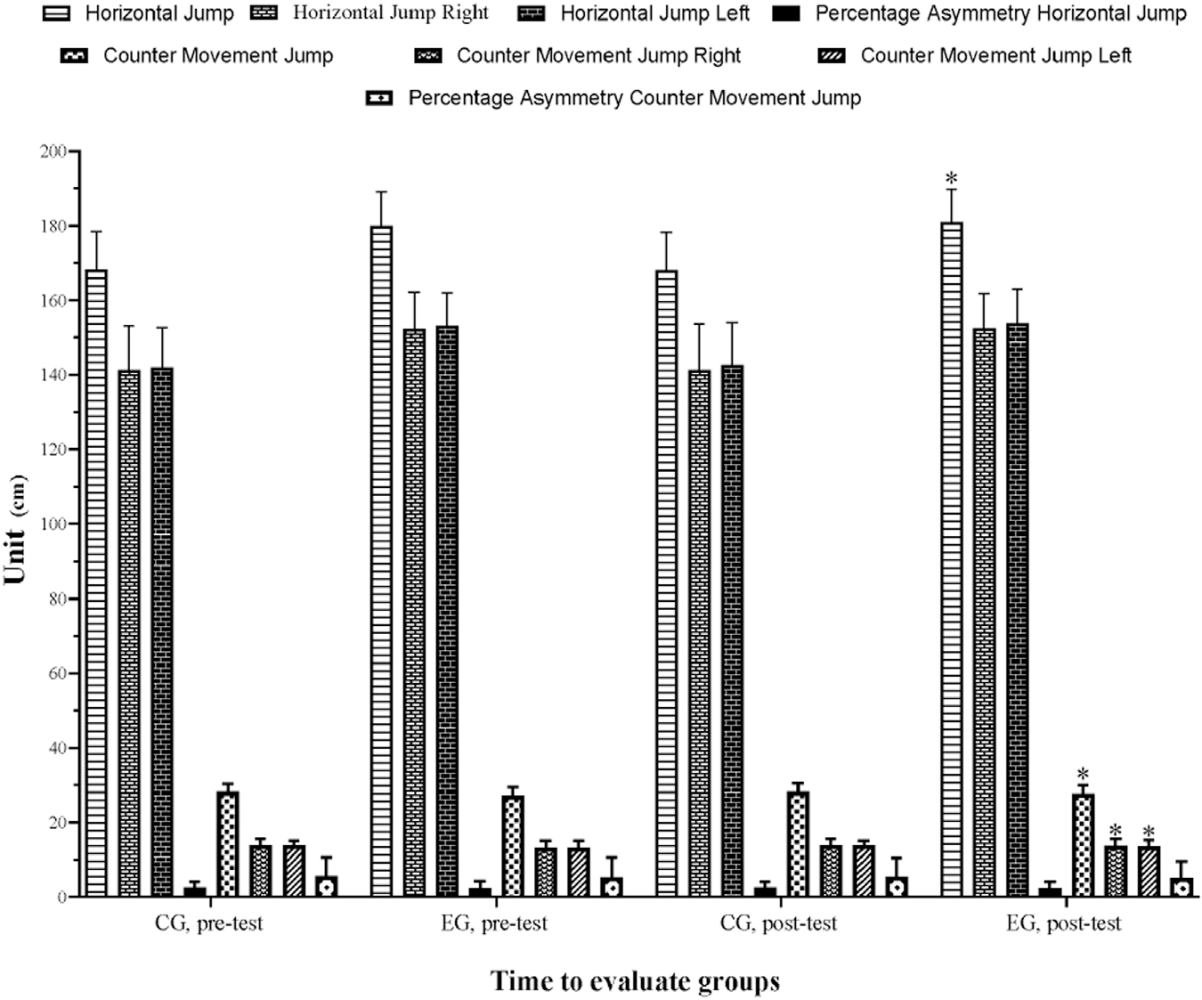
Variation in jump variables for control and experimental group.

**FIGURE 4:**
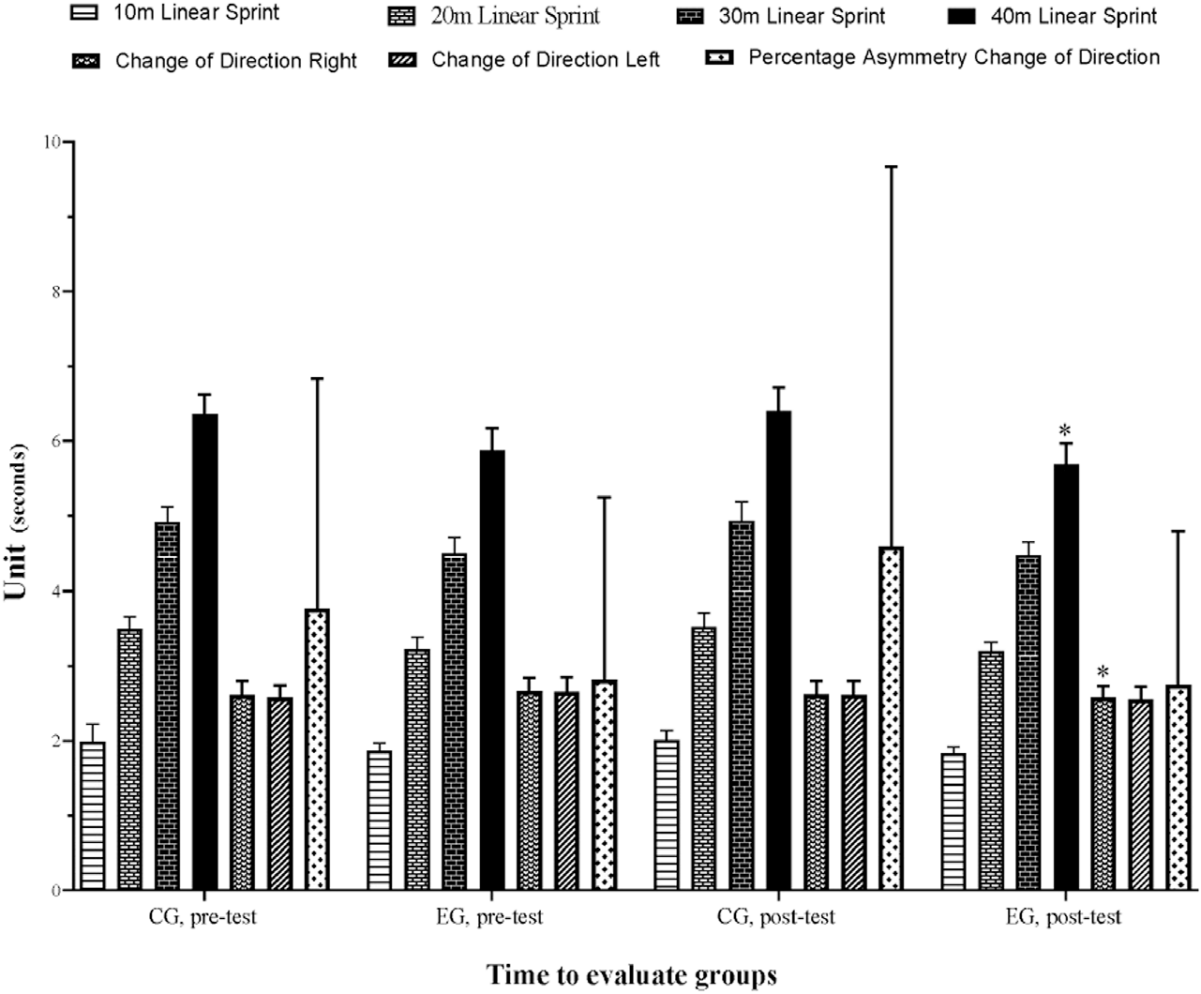
Variation in sprint and change of direction variables for control and experimental group.

The ANCOVA results showed significant group by time interactions for 10 m (*p* ≤ 0.001, f = 18.993, ηp^2^ = 0.352), 20 m (*p* ≤ 0.001, f = 10.345, ηp^2^ = 0.228), 30 m (*p* = 0.040, f = 4.534, ηp^2^ = 0.115), 40 m (*p* ≤ 0.001, f = 17.430, ηp^2^ = 0.332), COD right (*p* = 0.009, f = 7.653, ηp^2^ = 0.179), COD left (*p* = 0.009, f = 7.660, ηp^2^ = 0.180). When comparing the percent change from pre-to post-intervention in 40m, the independent groups *t*-test revealed a significant difference between, EG and CG (*p* ≤ 0.001). 40 m in the, EG increased by −3.01% compared to 0.68% in CG. Table 3 and Figure 3 and Figure 4 illustrate the percentage changes in sprint and COD variables between the pre- and post-test.

**TABLE 3:** Results of sprint and change of direction variables in control and experimental group.

Variables s)	CG (n = 20)				EG (n = 18)			
	Pre-intervention (mean ± SD)	Post-intervention (mean ± SD)	Pre-post (%)	ES (95% CI)	Pre-intervention (mean ± SD)	Post-intervention (mean ± SD)	Pre-post (%)	ES (95% CI)
10 m	1.98 ± 0.24	2.01 ± 0.13	1.51	0.15 (−0.47; 0.77) T	1.87 ± 0.10	1.83 ± 0.09	−2.11	−0.40 (−1.06;0.26) S
20 m	3.49 ± 0.17	3.52 ± 0.19	1.03	0.16 (−0.46; 0.78) T	3.22 ± 0.16	3.19 ± 0.12	−0.95	−0.20 (−0.86; 0.45) S
30 m	4.91 ± 0.21	4.93 ± 0.26	0.51	0.08 (−0.54; 0.70) T	4.50 ± 0.22	4.48 ± 0.18	−0.54	−0.09 (−0.75; 0.56) T
40 m	6.36 ± 0.26	6.40 ± 0.31	0.68	0.13 (−055.; 0.69) T	5.87 ± 0.30	5.69 ± 0.28	−3.01	−0.59 (−1.26; 0.08) M
COD R	2.61 ± 0.19	2.62 ± 0.19	0.19	0.05 (−057.; 0.67) T	2.66 ± 0.18	2.58 ± 0.15	−3.01	−0.46 (−1.12; 0.20) S
COD L	2.58 ± 0.16	2.61 ± 0.19	1.08	0.16 (−0.46; 0.78) T	2.65 ± 0.20	2.55 ± 0.17	−3.81	−0.51 (−1.18; 0.15) M
% As COD	3.77 ± 3.06	4.60 ± 5.07	22.14	0.19 (−0.43; 0.81) T	2.82 ± 2.43	2.75 ± 2.05	−2.58	−0.03 (−0.68; 0.62) T

CG, control group; EG: experimental group; SD, standard deviation; COD, change of direction; R, right; L, left; % As: percentage of asymmetry; ES, effect size; CI, confident interval; T, trivial; S, small; M, medium; *p< 0.05.

## 4 Discussion

This study aimed to determine whether a 10-week neuromuscular intervention improves physical performance and reduces functional asymmetries of the lower extremities in female football players. The main results were that there were no significant group by time interactions in ROM and percentage asymmetry tests. However, significant group by time interactions were observed for CMJ, HJ, 10 m, 20 m, 30 m, 40 m, and COD tests. The experimental group showed a significant increase in HJ (0.55%) compared to the control group (−0.07%). In addition, the experimental group also showed a significant increase in 40 m (−3.01%) compared to the control group (0.68%). These findings suggest that the intervention had a positive impact on lower body power and speed performance in the experimental group.

Decreased ankle dorsiflexion ROM has been pointed out as an essential risk factor for developing lower limb injuries (Kaufman et al., 1999), as it increases strain on the soleus and gastrocnemius muscles during running (Sasaki and Neptune, 2006) and increases dynamic knee valgus in landings (Fong et al., 2011). As mentioned above, lower extremity misalignments could decrease performance in key football actions, such as recently presented. The lack of significant group by time interactions in ROM test in the present study may be due to various factors, such as the duration and intensity of the neuromuscular training program, the athletes’ initial level of physical fitness, and the specific exercises used in the program. Evidence regarding the effects of neuromuscular interventions on dorsiflexion ROM is scarce. Previous studies (Noyes et al., 2013; Pardos-Mainer et al., 2019) indicated small ES (∼0.23 and 0.30, respectively), and a systematic review of post-ankle sprained subjects (Terada et al., 2013) pinpointed that static stretching plus proprioceptive and strengthening exercises may be the most effective stimulus. These studies suggest that longer duration and higher intensity of neuromuscular training programs than in the present study may be necessary to elicit significant improvements in ROM in female soccer players. However, the effectiveness of such programs may depend on various factors, and more research is needed to determine the optimal duration, intensity, and exercises in neuromuscular training programs for improving ROM in this population.

Straight sprinting is a key performance factor in professional football, as it has been identified as the most frequent action in goal-scoring situations (Faude et al., 2012). The results of the present study suggest that the intervention had a positive effect on sprint performance. The results of the significant post-hoc analysis for 10 m, 20 m, and 30 m sprints (*p* ≤ 0.001), indicate that the intervention had a greater impact on these variables in the experimental group compared to the control group. In this regard, Noyes et al. (Noyes et al., 2013) implemented a 6-week comparable training intervention on female football players aged 12–18 years, showing significant improvements (*p* = 0.02; ES = −0.14) in linear speed (37 m), whereas Pardos-Mainer et al. (2019) found similar results (*p* = 0.01; ES = −1.16) in adolescent female football players. Despite the age difference, both intervention protocols were very similar to ours and support the idea that neuromuscular training programs, which include strength, power and plyometric exercises, can be consistent in training methods with the intention to improve acceleration and short-distance sprint performance. In this sense, improvements in the power of the hip, knee and ankle extensors have previously been related to gains in sprinting ability (Morin et al., 2012), and it is also likely that the selection of exercises focusing on the horizontal stimulus has increased the chances of obtaining adaptations related to acceleration performance (González-García et al., 2019). On the other hand, Vescovi et al. (Vescovi and VanHeest, 2010) found a time effect in the speed improvements as the gains obtained in their study at week six disappeared at follow-up (week 12), underlining the importance of maintaining a minimum stimulus dose. These findings have implications for coaches and athletes seeking to improve sprint performance, as they suggest that a targeted intervention can be effective in enhancing these aspects of athletic performance. Further research is needed to identify the most effective components of such interventions and to determine the optimal duration and intensity of training needed to achieve maximum benefits in sprint performance.

Improving the speed of COD has become one of the main objectives of preparation programs (Vescovi and VanHeest, 2010). Of note, a recent investigation (Loturco et al., 2018) revealed that a higher linear speed is not necessarily related to better results in COD tests. The results of the significant post-hoc analysis for COD to the right and left indicate that the intervention had a greater impact on these variables in the experimental group compared to the control group. In the same way, a recent systematic review and meta-analyses (Liu et al., 2021) on the effects of a neuromuscular standardized warm-up protocol reported an overall improvement in COD tests in football players (ES = 0.87). In-season neuromuscular strength training intervention presented a –3.5% COD performance improvement after only 8 weeks (Panagoulis et al., 2020). Similar results were reported by Pardos-Mainer et al. after the combination of dynamic and isometric strength training showed a moderate effect (ES = −0.71) on COD performance (Pardos-Mainer et al., 2020). Plyometric training resulted in superior outcomes in COD tests, exhibiting a large effect (ES: -3.12) (Ramirez-Campillo et al., 2018). This may be attributed to the incorporation of vertical, horizontal, and unilateral jumps in these programs, which effectively enhanced COD performance. It is important to note that neuromuscular training programs can include a variety of exercises and techniques, such as plyometrics, balance and stability training, and agility drills, among others. Therefore, it is impossible to conclude if specific, or the combination of exercises in this intervention elicted the performance improvements. This also limits our ability to make direct comparisons with other studies. Nonetheless, the present results provide additional evidence supporting the effectiveness of neuromuscular training programs in improving COD performance.

The present study offers valuable insights into the impact of a neuromuscular training program on CMJ performance (ES = 0.23) in female football players, with only minor improvements observed in HJ (ES ≤ 0.10). These results are consistent with those reported in prior studies by Ozbar et al. (2014); Pardos-Mainer et al. (2019), which suggest that the nature of the power drills included in the neuromuscular program may have contributed to the observed outcomes, as vertical stimulus predominated over horizontal jumping tasks. Interestingly, the results of our study demonstrate that the neuromuscular training program had a specific effect on lower interlimb jump performance. This is supported by the significant post-hoc analysis observed for CMJ left and right. Although other studies (Gorostiaga et al., 2004; Wong et al., 2010) have demonstrated superior results in CMJ height (ES = –0.95) when applying longer explosive and plyometric strength programs to younger football players, the effect of neuromuscular interventions on the vertical jumping ability of female football players has been unclear in some (Noyes et al., 2013; Pardos-Mainer et al., 2019). Furthermore, a recent meta-analysis (Pardos-Mainer et al., 2021) compared the effects of plyometric versus strength training on essential factors of football performance. The results from this study highlight the importance of including high-speed exercises in the design of football conditioning programs, as plyometric training provides more significant benefits than strength training on vertical jump in female football players. Thus, incorporating a variety of exercises that promote explosive power and speed, rather than solely focusing on extra load exercises (Pedersen et al., 2019), may be a more effective strategy for improving vertical jump in female football players.

Historically, more significant interlimb asymmetry has been associated with lower athletic performance and increased risk of injury. However, recent studies show contradictory results (Bishop et al., 2018). Furthermore, Bishop et al. (2021) indicate that asymmetries are task-specific and that results may vary depending on the test performed. Focusing on analysing asymmetries and their relationship with performance, a recent systematic review with meta-analysis (Bettariga et al., 2022) confirms the importance of studies in which strength training interventions have been carried out with unilateral and bilateral exercises, plyometric work, balance and lumbopelvic stability. Despite our neuromuscular training program did not result in significantly different improvements in asymmetry tests results between the groups, the intra-group analysis showed that the experimental group has reduced ROM asymmetry by −3.97% (ES = −0.04), CMJ asymmetry by −2.37% (ES = 0.02), HJ asymmetry by −0.87% (ES = 0.01), and COD asymmetry by −2.58% (ES = 0.03). These results are reinforced in a previous review (Bettariga et al., 2022), in which slight to moderate effects on asymmetry reduction were observed across all interventions, but no significant differences were found for the HJ (ES: 0.22), the CMJ (ES = 0.53) and the COD (ES = 0.23). However, it is important to note that the lack of significance may be due to a variety of factors, such as the sample size, the specific measures used, and/or the duration and intensity of the training program. Finally, these results continue to confirm that the reduction of asymmetries and their relationship to improved performance should be further investigated.

### 4.1 Limitations

Of note, there exist a few limitations in the current study. Firstly, the small sample size prevented us from assessing differences between players’ positions. However, the primary objective of the present investigation was to analyse the overall effects of a neuromuscular training program on football performance variables and to determine whether the effects are position-specific, thus suggesting a potential area for future research. Furthermore, only high-level female football players were selected for the final analyses, so our results cannot be generalized to another level of performance or different team sports. Despite these considerations, the present research provides relevant data on the positive effects of the weekly application of strengthening programs with a neuromuscular emphasis on crucial performance variables of female football players.

## 5 Conclusion

The results of the present work emphasize that a 10-week neuromuscular training intervention may be a sufficient stimulus to improve physical fitness variables in high-level female football players. Significant improvements in COD tests and straight sprinting time were found after the intervention, while slight to trivial gains in jumping, ankle mobility, and interlimb symmetries were also registered. Therefore, players and coaches should be aware that the weekly inclusion of strength, power and dynamic balance exercises following a neuromuscular paradigm is useful for improving football fitness. Such gains can be achieved with an on-field resistance training program without supplemental materials to body mass.

## Data Availability

The original contributions presented in the study are included in the article material, further inquiries can be directed to the corresponding author.
